# Using Motor Imagery to Access Alternative Attentional Strategies When Navigating Environmental Boundaries to Prevent Freezing of Gait – A Perspective

**DOI:** 10.3389/fnhum.2022.750612

**Published:** 2022-03-29

**Authors:** Daniella How, Heiko Wagner, Michael Brach

**Affiliations:** Department of Movement Science, Institute of Sport and Exercise Sciences, University of Münster, Münster, Germany

**Keywords:** freezing of gait (FOG), motor imagery (MI), Parkinson’s disease (PD), attentional strategies, telemedicine

## Abstract

Freezing of gait can cause reduced independence and quality of life for many with Parkinson’s disease. Episodes frequently occur at points of transition such as navigating a doorway. Therapeutic interventions, i.e., drugs and exercise, do not always successfully mitigate episodes. There are several different, but not exclusive causes for freezing of gait. People with freezing of gait are able to navigate dynamic situations like stairways by utilizing a different attentional strategy to over-ground walking, but may freeze when passing through a doorway. The question is, is it possible to employ a special attentional strategy to prevent freezing at this point? Motor imagery allows for learning motor skills in absolute safety and has been widely employed in a variety of populations, including other neuro-compromised groups. Motor imagery is not studied in a homologous manner in people with Parkinson’s Disease, leading to conflicting results, but may have the potential to establish a different attentional strategy which allows a subject to mitigate freezing of gait episodes. This paper will identify and discuss the questions that still need to be answered in order to consider this approach i.e., can this population access motor imagery, can motor imagery alter the attentional strategy employed when moving through doorways, what is the best motor imagery approach for people with Parkinson’s Disease and freezing of gait, and what dosage is most effective, while briefly outlining future research considerations.

## Introduction

Between one third and 63% of patients with Parkinson’s Disease (PD) suffer from Freezing of Gait (FOG), which results in falls, decreased independence and reduced quality of life (QoL) (Rutz and Benninger, 2020; Silva-Batista et al., 2020). FOG is characterized by an abrupt, erratic gait interruption (Peterson et al., 2014; Pozzi et al., 2019) despite the person intending to move forward (Nutt et al., 2011). This can result in hesitation starting a movement, stopping during turns (Plotnik and Hausdorff, 2008) and trembling in place (Moore et al., 2008; Nonnekes et al., 2019). While medication with levodopa has been shown to be partially effective, FOG is still sporadically observed when patients are in the clinical “ON”-medication state (Schaafsma et al., 2003a). FOG episode (Lees, 1989; Pozzi et al., 2019; Silva-Batista et al., 2020) severity correlates with disease progression (Paul et al., 2018). People with PD are considered to be in an ON-medication state when levodopa medication is effective in alleviating motor symptoms and in the OFF-medication state when there is not enough levodopa remaining in the brain to relieve motor symptoms sufficiently (Lees, 1989). Falls frequency is associated with FOG both in OFF-medication and ON-medication states but far greater in the OFF-medication state (Schaafsma et al., 2003b). Other non-invasive treatments which alleviate FOG severity include exercise rehabilitation programs (Cucca et al., 2016; Silva-Batista et al., 2020) but rehabilitation gains are not often retained (Lees, 1989; Gilat et al., 2021). If both rehabilitation and medication are not successfully alleviating FOG severity, then further treatment options should be explored.

Because FOG can occur during ON-medication states (Lewis and Barker, 2009), it may not be solely attributed to depleted dopamine (Schaafsma et al., 2003a; Lewis and Shine, 2016). Current theories as to the cause of FOG include:

(i) Poor conflict resolution between anticipatory postural adjustments (APAs) with stepping patterns between sensory motor area (SMA) and motor cortex (Schaafsma et al., 2003a). The person is stuck in a ready state;
(ii) Disconnection between basal ganglia (BG) and the SMA, causing interruption in internal cueing of learnt actions. This is further complicated by competition from the increased excitatory output of the subthalamic nucleus (STN) and other centers for motor, cognitive, and limbic cortical areas. BG fire synchronously and inhibition occurs in brain stem areas resulting in FOG. This theory is supported both by dopamine reducing FOG and by cueing which bypasses the caudate nucleus, thalamus and prefrontal cortex motor loop, allowing motor activity (Lewis and Barker, 2009);
(iii) Visuo-spatial judgment failures resulting from poor communication between the prefrontal cortex and the BG (Kostic et al., 2012);
(iv) Executive function as a result of poor communication between BG and the frontal lobe, especially evident in dual task scenarios where overload from competing demand interrupts communication (Shine et al., 2013a,b; Cucca et al., 2016; Marquez et al., 2020).

An earlier theory proposes a disruption of supraspinal controls to the central pattern generators (CPGs) (Plotnik and Hausdorff, 2008; Marquez et al., 2020). These causes may not be exclusive of each other.

Freezing of Gait is experienced by individuals with PD at environmental boundaries such as doorways, or crossing roads, when in tight spaces, and when facing an obstacle such as furniture or trip hazards such as uneven flooring (Ramos et al., 2020). However, people with PD report fewer FOG episodes when navigating obstacles such as stairways where specific attentional strategies are used, e.g., subjects may be able to focus on keeping each step even following the step treads (Rutz and Benninger, 2020) or when using a pedestrian cross walk following evenly spaced lines (Ramos et al., 2020). Could a person who experiences FOG learn and utilize a similar strategy to regulate their gait without freezing at an environmental obstacle or boundary?

In healthy individuals, motor control is not just regulated by CPGs and the brainstem but refined with cortical commands (Pozzi et al., 2019). The neuronal circuits that comprise CPGs which result in rhythmic movements such as walking are always active, but modulated by higher brain centers (Marder and Bucher, 2001; Behrendt et al., 2013, 2014). The cerebral cortex modulates rhythms that originate in spinal networks. In the PD brain, the depletion of dopamine may result in not enough to act as a neurotransmitter. Competition is created between complementary neural circuits, both inhibitory and excitatory (Lewis and Barker, 2009) which leads to FOG.

Rehabilitation exercises developed specifically to improve FOG symptoms show benefits (Gilat et al., 2021), but as those benefits are not retrained, would need to be continuously practiced to preserve the improvement. A safe, but effective adjunct therapy could be utilized to facilitate this practice independently, without the need for additional support by staff (Heremans et al., 2012). In this article the authors will propose why Motor Imagery (MI) could support standard rehabilitation practice to prevent FOG at environmental boundaries.

## Motor Imagery as a Therapy for Parkinson’s Disease Patients to Support Motor Control

Motor imagery is the mental rehearsal of an action without its actual physical performance. It can be “performed” in the first person or “viewed” as a third person. MI can be visual in nature or kinesthetic (Saimpont et al., 2013). MI has been used successfully in healthy adults, especially in sports, for some time (Saimpont et al., 2013). One of the draw backs is it can be mentally demanding (Abbruzzese et al., 2015) but it can be performed seated as a visualization only without safety risks, and, once the skill is learnt, can be practiced independently without a therapist present (Heremans et al., 2012). This would not replace other rehabilitation practices but adjunct them (Slimani et al., 2016; Da Nascimento et al., 2019). MI is now being used in training for healthy older adults as an adjunct to physiotherapy. Studies are conflicting, but research currently supports the idea that MI can improve the outcomes of therapeutic interventions in older populations (Saimpont et al., 2013). It is believed to preserve or stimulate the forward planning neural pathways (Ashley Fox, 2013). MI shows the most benefit in healthy elderly adults when in the third person, supported by auditory cues and kinesthetic in nature (Saimpont et al., 2013). Importantly, the pathways used in MI are partially the same as those used in motor execution (Snijders et al., 2011), specifically the SMA, premotor cortex, primary motor cortex, posterior parietal regions (e.g., the inferior and superior parietal lobes), the BG and cerebellum (Moran and O’Shea, 2020).

Motor imagery alone can cause a significant improvement in muscle force, as well as increasing the motor-activity related cortical potential of healthy older adults (Jiang et al., 2016). Mouthon et al. (2015) showed that during MI, cortico-spinal excitability is increased in healthy young adults during mental training of balance tasks, as evidenced by motor evoked potential facilitation. In healthy subjects, brain plasticity can be measured after MI, and motor performance is improved (Debarnot et al., 2014).

Traditional gait and balance therapy is usually delivered in an explicit teaching method. Explicit learning is a complex learning method requiring a high cognitive load, using working memory. As people age, and with the impact of disease, the ability to learn through explicit methods deteriorates. This is especially true in dual-task situations (Abbruzzese et al., 2015). People are expected to integrate and memorise instructions in traditional motor learning (Rutz and Benninger, 2020). This style of explicit learning is not retained in people with brain lesions in the motor pathways such as PD (Rutz and Benninger, 2020).

An important consideration for any clinician wishing to consider a patient for this therapy is their cognitive decline. People with PD may exhibit cognitive decline which varies with the stage and time of onset of the disease (Ding et al., 2015). Salient to gait rehabilitation are losses in executive function, short-term working memory and visual-spatial memory. Early cognitive impairment is difficult to diagnose. There is no clear pattern to the decline and the decline can only be assessed on an individual basis and is unique to each person (Ding et al., 2015). The gross progression of the pathology of PD will eventually include atrophy of several brain regions (Ding et al., 2015), some of which we can surmise could affect the person’s ability to engage in MI and overall gait rehabilitation.

The advantage of MI is that it does not require learning (Snijders et al., 2011), it uses working memory efficiently, and allows for greater training endurance (Debarnot et al., 2014; Moran and O’Shea, 2020). Gait re-education and the creation of new attentional strategies through means such as MI should allow for greater retention of motor learning in people with PD (Mirelman et al., 2013). MI can be mentally fatiguing (Abbruzzese et al., 2015) and the authors suggest that using MI as an intervention (Podda et al., 2020) should be considered on a case-by-case basis, and that an appropriate dose is considered for that individual. It may not always be an appropriate intervention with this condition.

There are however, a number of questions that need to be carefully considered before pursuing MI as a possible treatment modality for FOG. These are:

## Can Motor Imagery Supplement Gait Rehabilitation Therapy for People With Parkinson’s Disease and Freezing of Gait?

### Can the General Parkinson’s Disease Population Access Motor Imagery?

There is continued debate if people with PD can access MI, but studies have used inconsistent protocols with poor follow-up (Da Nascimento et al., 2019). The study of Abraham et al. (2018) showed promising results with improvements for the experimental group in motor symptoms, balance, physical activity and others. Their study was, however, poorly controlled. The MI group received an intensive in-person movement session whereas the control group received literature and an exercise video to perform at distance. They were asked to submit a video of exercise themselves, but the study does not describe how many completed the task. Another study indicated that MI was equally effective as relaxation (Tamir et al., 2007) therapy and that both improved gait in people with PD (Braun et al., 2011). They did not specifically address FOG. Tamir et al. (2007) compared conventional physical therapy with a combined motor imagery and conventional training approach and found that the latter was more effective, especially in reducing bradykinesia. The combined approach group showed “significantly faster performance of movement sequences than the control group.” Further research is needed to either prove or disprove the success of MI with people with PD.

### Can the Freezing of Gait Parkinson’s Disease Population Access Motor Imagery?

Studies show that people with PD can access MI. The bradykinesia they demonstrate in gait is also apparent in their MI. People with FOG from PD visualize their MI more slowly than healthy controls and more slowly than people with PD without FOG (Cohen et al., 2011). When people with PD imagine their MI activity passing obstacles that cause them FOG, they experience an incongruency with time when compared with people with PD that do not have FOG imagining traversing the same obstacles (Cohen et al., 2011). Simply put, they are delayed in the imagined activity where they would be delayed in the same motor activity. This may be linked with the same deterioration in the motor circuits related to FOG. Overground walking has been observed to correlate with imagined walking times in PD for those with FOG and without (Peterson et al., 2014). Both these aforementioned observations have been under MRI scanning and not in a gait re-education program or with external cueing.

### Can Motor Imagery Alter the Attentional Strategy Employed When Moving Through Doorways?

This is not yet known and requires the greatest research. Moran and O’Shea (Moran and O’Shea, 2020) discuss how MI can make the neural systems more efficient, possibly by changing how motor information is processed in the working memory, but admit that not enough is known about how the process works. The dorsolateral prefrontal cortex is active during MI which results in better working memory. Lewis and Shine (2016) also discuss that “global neuronal efficiency” should help alleviate FOG but that the underlying mechanisms to ensure such efficiency are not yet known. None of these studies answer if MI can improve gait learning in people with PD.

Cohen et al. (2011) established that people with PD and FOG demonstrate a mismatch between the motor execution and their motor imagery when approaching and walking through doorways. People with PD and FOG in this experiment slowed more than people with PD but without FOG as they approached a doorway. However, when they imagined the same activity, they did not slow more than their experimental counterparts. They did not demonstrate a visual mismatch or an altered body schema when judging a door width. The suggested causes were an impairment in motor execution or a poor understanding of their gait impairment. If it is an impairment in motor execution, it suggests that training to improve APAs accessed when walking toward and through a doorway might improve the speed. It is not known if MI can improve APAs for people with FOG and further research is required in this subject area.

### What Would Be the Best Approach of Motor Imagery in Rehabilitation Therapy?

In MI, older individuals show a stronger response to auditory cues (Hovington and Brouwer, 2010). Cueing for MI should support the kinesthetic or “visual” nature of the imagined scenario and can be in the first or third person (Abraham et al., 2018). External auditory cueing can be combined with kinesthetic MI to produce tailored rehabilitation programs for individuals (Cohen et al., 2011).

Any MI practice requires that the patient be assessed for a preference in their MI learning style. This can be assessed using a motor imagery questionnaire (Saimpont et al., 2013; Da Nascimento et al., 2019). Therapists need to be consistent with the practice and cueing style (Piccoli et al., 2018).

### What Would Be the Duration, Frequency and Dose of a Suitable Motor Imagery Intervention?

A review of MI show incongruous durations and intervention times for MI studies in older populations. The findings show results in older research participants are possible in under a month with doses of less than 20 min long, 3 to 4 times per week (Schuster et al., 2011). The authors of this paper concede that a longer intervention duration may be required for individuals with FOG, as motor learning of all types worsens with PD (Olson et al., 2019).

One of the benefits of MI is the potential for the passive nature of the practice. It can safely be performed in sitting with no danger to the subject. This may allow the possibility of developing an online, patient specific therapeutic intervention. This might lower related costs to both therapist and patient (Heremans et al., 2012). During the Covid-19 pandemic, telemedicine has become an all-important way for patients to access therapy. Safety is always a consideration and limitation in what can be executed virtually (Middleton et al., 2020). It is not known if MI could be supervised virtually. This intervention is always an adjunct to standard physical therapies (Saimpont et al., 2013).

## Discussion and Conclusion

Subjects with FOG experience a worsened QoL and risk falls through the sudden interruption of gait. Although much has been discovered about the underlying causes, no treatment to date has been completely successful in eliminating FOG. FOG often occurs at specific moments or places, such as environmental boundaries e.g., a person with FOG may get “stuck” at the threshold of a doorway. FOG rehabilitation exercises show benefits, but those benefits are not retained without ongoing intervention. What if the person with FOG could independently continue those exercises in absolute safety inside their own minds, thus continuing to benefit from improved mobility without a therapist present–MI?

Because studies of MI in FOG are not homologous in design, it is difficult to compare studies to have a clear understanding if MI in PD is an effective treatment to reduce FOG and how best to apply it. The drawbacks are that MI may cause fatigue and that it has been difficult to assess in PD subjects. For the therapist some treatment factors are unknown, such as an effective dose. An individual may not be able to fully benefit due to cognitive decline. It also requires practice on the part of the therapist to teach the technique consistently. The proposed benefits are that MI of an action uses many of the same neural pathways as the physical movement, has the potential to produce motor neuron plasticity, is an efficient way to learn and has shown substantial treatment benefits in other populations. It should not be disregarded without complete study and a larger body of comparable and well controlled research should be carried out to establish if MI can mitigate FOG at environmental boundaries.

## Data Availability Statement

The original contributions presented in the study are included in the article/supplementary material, further inquiries can be directed to the corresponding author/s.

## Author Contributions

DH had the idea for the article with support from MB and drafted the article with inputs from HW and MB. DH and HW performed the literature search. All authors critically revised the work.

## Conflict of Interest

The authors declare that the research was conducted in the absence of any commercial or financial relationships that could be construed as a potential conflict of interest.

## Publisher’s Note

All claims expressed in this article are solely those of the authors and do not necessarily represent those of their affiliated organizations, or those of the publisher, the editors and the reviewers. Any product that may be evaluated in this article, or claim that may be made by its manufacturer, is not guaranteed or endorsed by the publisher.
